# Sleeplessness and incident diabetes above the Arctic circle: a secondary analysis of cohort data from the Tromsø Study

**DOI:** 10.1136/bmjph-2023-000644

**Published:** 2024-01-10

**Authors:** Nick Chindanai Uthaikhaifar, Olena Iakunchykova, Sarah Cook, Charlotte Warren-Gash

**Affiliations:** 1 London School of Hygiene & Tropical Medicine, London, UK; 2 Center for Lifespan Changes in Brain and Cognition, Department of Psychology, University of Oslo, Oslo, Norway; 3 School of Public Health, Imperial College London, London, UK; 4 Faculty of Epidemiology & Population Health, London School of Hygiene & Tropical Medicine, London, UK

**Keywords:** Epidemiology, Public Health, Community Health

## Abstract

**Introduction:**

Circadian misalignment and sleep quality are intertwined processes that are both associated with diabetes. The association between sleep quality and incident diabetes has not been previously investigated in populations living at polar latitudes who experience extreme seasonal daylight variation and may be at greater risk of circadian misalignment. Using data from adult residents of Tromsø, Norway, this study investigates the association of poor sleep quality, as indicated by self-reported sleeplessness, and incident diabetes above the Arctic circle.

**Research design and methods:**

Secondary analysis of cohort data from the Tromsø Study. The study cohort consists of adults who attended both the fourth (Tromsø4) and seventh (Tromsø7) surveys conducted in 1995 and 2016, respectively. Only individuals with complete data were included. Multivariable logistic regression was used to examine the association between sleeplessness measured in Tromsø4 and incident diabetes measured in participants followed up to Tromsø7, adjusted for other diabetes risk factors.

**Results:**

Among 10 875 individuals (mean 41 years of age at baseline, 53.6% women), 21.2% (n=2302) reported experiencing sleeplessness at baseline. Diabetes incidence risk over follow-up (20 years) was 7.2% (n=784); incidence risk among individuals reporting sleeplessness was 8.8%, compared with 6.8% among unexposed individuals. After adjustment, sleeplessness-exposed individuals in the study cohort were found to have 23% greater odds (OR_adj_ 1.23, 95% CI 1.03 to 1.47, p=0.022) of incident diabetes.

**Conclusions:**

Sleep quality is associated with incident diabetes in a population living above the Arctic circle. The direction and strength of association is consistent with findings from other geographical regions.

**WHAT IS ALREADY KNOWN ON THIS TOPIC**

**WHAT THIS STUDY ADDS**

- Among residents of Tromsø, northern Norway, sleeplessness is associated with 23% greater odds of incident diabetes over a 20-year follow-up period.

**HOW THIS STUDY MIGHT AFFECT RESEARCH, PRACTICE OR POLICY**

- Our results demonstrate that the association between poor sleep quality and incident diabetes in an Arctic population is similar in direction and strength to findings from populations living at lower latitudes. This consistency adds further support for designating sleep quality as an independent risk factor for diabetes and a potential target for prevention programmes.

## Introduction

 Within Norway, 5% of the overall population (approximately 245 000 individuals) are estimated to have diagnosed diabetes mellitus based on diabetic drug prescription data and diabetes is the seventh-leading cause of morbidity measured by years lost to disability.^1^ Although the incidence rate has stabilised,^2^ diagnosed diabetes prevalence continues to increase, representing a significant burden on the Norwegian healthcare system.^1^

There is a growing evidence that poor sleep quality, which has been found to also be increasing in prevalence in Norway,^3^ is a significant, independent risk factor for diabetes; a systematic review and meta-analysis covering 11 cohort studies up to 2013 found that poor sleep quality was associated with a 38% increase in diabetes risk after adjustment for other diabetes risk factors.^4^ Further studies have also found positive associations between poor sleep quality and diabetes,^5 6^ although the effect of sex on this association remains unclear as previous studies^7^,^9^ in Scandinavian populations found an association only among men, while studies in western Europe^10 11^ and Japan^12^ reported no difference in association by sex.

Hypothalamic–pituitary–adrenal (HPA) axis hyperactivation^13 14^ and hormonal dysregulation of leptin^15^ due to disturbed sleep have been proposed as possible biological mechanisms leading to diabetes. Experiments have also demonstrated that sleep quality^16^ and glycaemic control^17^ are both directly impaired by misalignment in timing between the endogenous circadian cycle and exposure to environmental light. The circadian rhythm has also been shown to be sensitive to seasonal daylight variation,^18^ with one study noting a high incidence of circadian misalignment among individuals wintering in Antarctica.^19^ However, the precise interaction between sleep quality, circadian misalignment and seasonal daylight variation in the development of diabetes remains unclear.^20^

In this context, this study aims to examine the association between sleep quality and incident diabetes among participants of the Tromsø study, who experience extreme seasonal daylight variation and may be at greater risk of circadian misalignment. To the authors’ knowledge, this study is the first such epidemiological investigation among residents of Northern Norway and, more broadly, any Arctic population.

## Research design and methods

The Tromsø Study is an ongoing population-based cohort study, which aims to enrol adult residents of Tromsø municipality in Northern Norway.^21^ Seven surveys have been completed since the study began in 1974; this paper analyses data from the fourth (Tromsø4) and seventh (Tromsø7) surveys conducted in 1994–1995 and 2015–2016, respectively. At each survey, participants received two self-administered questionnaires collecting data on demographic, health history/status and behavioural data. Participants also attended a physical examination visit where anthropometric measurements and blood samples were collected. For Tromsø4 only, laboratory measurement of glycated hemoglobin (HbA1c) was limited to a subsample of participants in an extended survey consisting of all participants aged 55–74 and between 5% and 10% of participants aged 25–54 and 75–85. Data from intermediate surveys, Tromsø5 (2001) and Tromsø6 (2007–2008), were not used as only a subset of the population was invited to participate in these surveys (see online supplemental figure 1).

### Study participants

All residents aged 25 or older at enrolment (born before 1970) were invited to participate in Tromsø4; 27 158 individuals agreed to take part in the study, resulting in a 77% participation rate among the target population excluding individuals who had moved or died prior to the study. At Tromsø7, all residents aged 40 or older at enrolment were invited to participate; 21 083 individuals agreed to take part in the study (65% participation rate).

**Figure 1:**
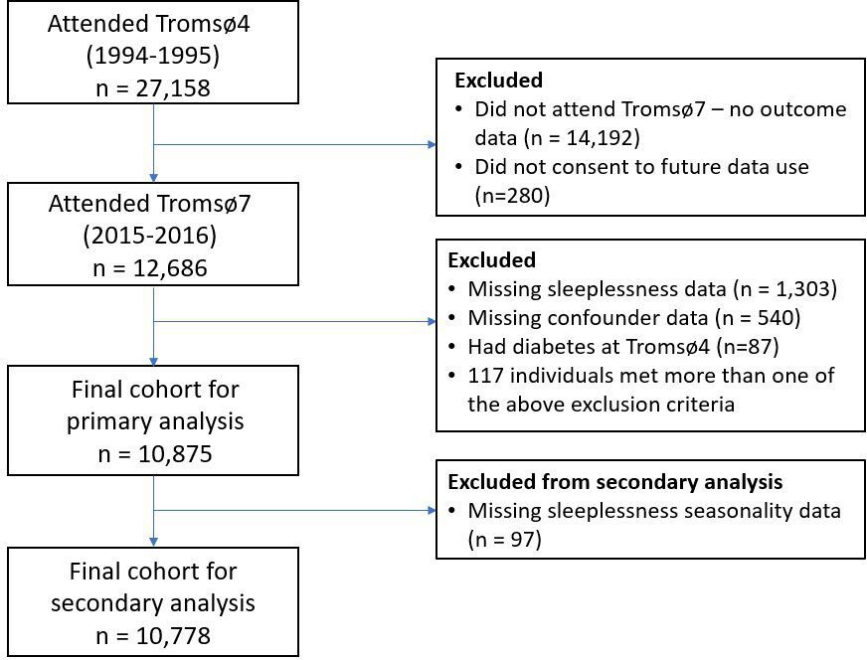
Study participant exclusion flow from Tromsø4 baseline.

Of the 27 158 participants enrolled in Tromsø4, 12 686 (46.7%) also participated in Tromsø7 and consented to have their data used in further research. Of this total, data on sleeplessness exposure, diabetes outcome and included confounders were complete for 10 945 individuals and, after further excluding those with diabetes at baseline (n=70), 10 875 remained for primary analysis (figure 1). 97 participants reported experiencing sleeplessness at least 1–2 times a month but did not provide information on sleeplessness seasonality and were excluded from all secondary analyses of season-specific sleeplessness exposure (n=10 778).

### Exposure measurement

Exposure data on self-reported sleeplessness was collected in Tromsø4 by asking participants ‘How often do you suffer from sleeplessness?’. Possible response options were: ‘never or just a few times a year’, ‘1–2 times a month’, ‘approximately once a week’ and ‘more than once a week’.

Sleeplessness data were dichotomised as a binary variable for the primary analysis. Individuals who selected the least frequent response of ‘never, or just a few times a year’ were classified as not exposed (0) and all other responses were classified as exposed (1). Dichotomisation was required to ensure a sufficient number of cases in the exposed and unexposed groups for subsequent statistical analysis. Sleeplessness exposure categorised per original response options was also retained for sensitivity analyses to contextualise the results from the primary binary exposure variable.

A secondary categorical sleeplessness variable that reflects the impact of seasonal variation in environmental daylight exposure on sleep quality was created from the binary primary exposure variable by further stratifying participants in the exposed (1) group based on their answer to the question: ‘If you suffer from sleeplessness, what time of the year does it affect you most?’. The following possible responses for sleeplessness seasonality were used as the categories for this secondary analysis: ‘no particular time of the year’, ‘especially during the polar night’, ‘especially during the midnight sun season’ and ‘especially in spring and autumn’.

Data for the following covariates were also available: age, sex, body mass index (BMI), daily smoking, time performing hard physical activity, frequency of binge drinking, highest level of education attained, family history of diabetes, hypertension and dyslipidaemia. Values for all covariates were obtained from data collected at Tromsø4, except for education and family history of diabetes which were obtained from Tromsø7, as these variables represent lifelong exposures and more recent data may provide the most accurate reflection of exposure. Participants’ hypertension and dyslipidaemia status were derived from blood pressure and lipid measurements at Tromsø4, respectively. Hypertension was defined based on the mean systolic and diastolic blood pressure of the second and third measurements made during participants’ physical examination. Participants with systolic pressure above 140 mm Hg or diastolic pressure above 90 mm Hg were defined as having hypertension.^22^ Dyslipidaemia status was defined as having a non-high density lipoprotein cholesterol (non-HDL-C) level of 4.3 mmol/L or greater, based on dyslipidaemia management guidelines by the Canadian Cardiovascular Society.^23^ Non-HDL-C was calculated as the difference between total cholesterol and HDL-C, both measured from participants’ non-fasting blood samples.

### Outcome measurement

A study participant was deemed to have the outcome of interest (incident diabetes mellitus) if at least one of the following applies: (1) self-reports insulin or non-insulin blood glucose-lowering drug usage, (2) self-reports currently having diabetes and (3) HBA1c level of 6.5% (48 mmol/mol) or greater as measured from a collected blood sample.

Data on self-reported diabetic status and antidiabetic drug use were collected in both Tromsø4 and Tromsø7; Tromsø4 data were used to exclude individuals with existing diabetes at baseline, while Tromsø7 data were used to identify incident diabetes cases. The phrasing of the questions on diabetes did not differ between the two waves although a separate, additional response option to specify previous (non-current) diabetic status was available for Tromsø7 that was not included for Tromsø4. Antidiabetic drug use was defined as using either insulin or other blood glucose-lowering drugs (A10A and A10B coded drugs per Anatomical Therapeutic Chemical classification, respectively).

HbA1c levels were measured from participants’ blood samples collected during their physical examination. These data were collected for 27% of Tromsø4 participants and 99% of Tromsø7 participants; only one HbA1c measurement was conducted for each participant. HbA1c level was used to determine participants’ diabetic status at baseline for cases with available data for this variable; the diabetic status of individuals with no HbA1c data was evaluated based only on self-reported data (antidiabetic drug use and diabetic status).

### Statistical analyses

The distribution of binary and categorical covariates was examined by tabulation in the overall cohort and each of the binary sleeplessness exposure groups. Continuous variables were summarised by the mean value and SD. Potential associations between sleeplessness and each categorical covariates were assessed by Pearson’s χ^2^ test. The unadjusted association between each variable and diabetes outcome quantified as an OR, was examined by univariable logistic regression.

A minimally adjusted model accounting for only age and sex was initially constructed as these variables were considered to be strong a priori confounders. A directed acyclic graph of assumed causal associations between all available variables was constructed (see online supplemental figure 2) to inform the selection of confounders. On this basis, the minimum sufficient adjustment set of confounders included in the final, fully adjusted logistic regression model was: BMI, family history of diabetes, education, daily smoking, time performing hard physical activity, hypertension and dyslipidaemia status.

Additional logistic regression models that include interaction terms were constructed to assess whether the effect of sleeplessness on incident diabetes is modified by sex or age. To ensure a sufficient number of participants in each stratum and avoid data sparsity issues, participants were grouped into the following age bands: <30, 30–39, 40–49, 50–59 and >60. Evidence of effect modification was assessed using the likelihood ratio test (LRT) comparing models with interaction terms specified to those without. A modified version of the final model that includes age as a categorical variable instead of a continuous variable was used as the comparator model for testing interaction by age.

The stability of the SE of the primary exposure coefficient was monitored at each step during model construction to screen for collinearity issues. Linearity of effect was not assumed for ordinal categorical covariates; these variables were included as simple categorical variables in the final model.

For secondary analyses, the same minimally and fully adjusted logistic regression models as described above were respecified with the secondary, season-specific sleeplessness exposure variable instead of the binary sleeplessness exposure variable. A sensitivity analysis was conducted in which sleeplessness was modelled as a categorical variable with exposure classified by the original survey question response options instead of as a binary exposure.

### Patient and public involvement

The participants were not involved in the design, conduct or reporting of this secondary data analysis. However, this study is included in the list of ongoing research projects based on the Tromsø study published on the Tromsø study main website and we hope that publication of the results in a peer-reviewed journal will facilitate further dissemination of the findings.

## Results

### Descriptive statistics

A larger proportion of individuals lost to follow-up (did not attend Tromsø7) did not perform any hard physical activity, smoked daily, had high BMI, hypertension and dyslipidaemia compared with those included in the primary analyses. Self-reported sleeplessness was more common among individuals lost to follow-up (29.4%) than in the study cohort (21.3%), providing strong evidence (p<0.001, χ^2^ test) that sleeplessness differed by lost to follow-up status (see online supplemental table 1). Diabetes outcome data were unavailable for individuals who did not attend Tromsø7. Although HbA1c data were only collected from 27% of all Tromsø4 participants, using this data when available to determine diabetic status at baseline resulted in the exclusion of seven baseline cases that would not have been identified using only self-reported diabetic status or antidiabetic drug usage at baseline.

The 10 875 individuals included in the primary analysis were aged between 25 and 76 at baseline (as of 31 December 1994), with mean age of 41 years (see table 1.) Women make up a larger proportion (53.6%) of the cohort than men. Approximately one-fifth (21.2%) of participants reported experiencing sleeplessness at least once per month at baseline. χ^2^ tests yielded strong evidence (p<0.001) of association between sleeplessness and age, sex, daily smoking, dyslipidaemia, education, time performing hard physical activity and binge drinking among study participants; there was some evidence (p=0.03) for an association between sleeplessness and hypertension. BMI and family history of diabetes were not observed to be associated with sleeplessness.

**Table 1:** Summary of demographic and behavioural variables by self-reported sleeplessness at enrolment

Characteristic at baseline	Total	Sleeplessness		P value*
		Less than once per month (unexposed)	At least once per month (exposed)	
	n=10 875	n=8573 (78.8)	n=2302 (21.2)	
Age (years)				
Mean (SD)	41.1 (9.7)	40.8 (9.5)	42.5 (10.3)	
Sex				<0.001
Female	5830 (53.6)	4438 (51.8)	1392 (60.5)	
Male	5045 (46.4)	4135 (48.2)	910 (39.5)	
BMI				0.355
<22	2436 (21.5)	1832 (21.4)	530 (23.0)	
22–23.9	2582 (22.8)	1987 (23.2)	510 (22.2)	
23.9–25.6	2308 (20.4)	1773 (20.7)	456 (19.8)	
25.6–28	2189 (19.4)	1661 (19.4)	437 (19.0)	
>28	1786 (15.8)	1320 (15.4)	369 (16.0)	
Blood pressure				
Systolic (mm Hg), mean (SD)	128.9 (15.5)	128.9 (15.2)	128.9 (16.4)	
Diastolic (mm Hg), mean (SD)	75.6 (10.9)	75.5 (10.7)	76.0 (11.4)	
Hypertension (Systolic blood pressure >140 mm Hg or Diastolic blood pressure >90 mm Hg)				0.034
No	8588 (79.0)	6807 (79.4)	1781 (77.4)	
Yes	2287 (21.0)	1766 (20.6)	521 (22.6)	
Blood lipids				
Total cholesterol (mmol/l), mean (SD)	5.8 (1.2)	5.8 (1.2)	5.9 (1.2)	
Triglycerides (mmol/l), mean (SD) (n=10 869)	1.4 (1.0)	1.4 (0.9)	1.5 (1.0)	
HDL (mmol/l), mean (SD)	1.5 (0.4)	1.5 (0.4)	1.5 (0.4)	
Dyslipidaemia (Non-HDL cholesterol≥4.3 mmol/L)				<0.001
No	5908 (54.3)	4743 (55.3)	1165 (50.6)	
Yes	4967 (45.7)	3830 (44.7)	1137 (49.4)	
Smoke daily				<0.001
No	7261 (66.8)	5870 (68.5)	1391 (60.4)	
Yes	3614 (33.2)	2703 (31.5)	911 (39.6)	
Hard physical activity				<0.001
None	4194 (38.6)	3228 (37.7)	966 (42.0)	
<1 hour/week	2697 (24.8)	2114 (24.7)	583 (25.3)	
1–2 hours/week	2679 (24.6)	2159 (25.2)	520 (22.6)	
3≥hours/week	1305 (12.0)	1072 (12.5)	233 (10.1)	
Binge drinking (past 12 months)				<0.001
Not at all in past year	3555 (32.7)	2809 (32.8)	746 (32.4)	
A few times	4402 (40.5)	3573 (41.7)	829 (36.0)	
1–2 times a month	2073 (19.1)	1575 (18.4)	498 (21.6)	
1–2 times a week	525 (4.8)	384 (4.5)	141 (6.1)	
Three or more times a week	36 (0.3)	20 (0.2)	16 (0.7)	
Missing	284 (2.6)	212 (2.5)	72 (3.1)	
Highest education level				<0.001
Primary/partly secondary education	3074 (28.3)	2321 (27.1)	753 (32.7)	
Upper secondary education	3259 (30.0)	2604 (30.4)	655 (28.5)	
Tertiary education, <4 years	2021 (18.6)	1637 (19.1)	384 (16.7)	
Tertiary education, ≥4 years	2521 (23.2)	2011 (23.5)	510 (22.1)	
Family history of diabetes				0.687
Yes	3557 (32.7)	2796 (32.6)	761 (33.1)	
No	7318 (67.3)	5777 (67.4)	1541 (66.9)	
Sleeplessness seasonality				<0.001
No particular time of the year	4392 (40.4)	3184 (37.1)	1208 (52.5)	
Especially during the polar night	1775 (16.3)	1082 (12.6)	693 (30.1)	
Especially during the midnight sun season	367 (3.4)	257 (3.0)	110 (4.8)	
Especially in spring and autumn	381 (3.5)	187 (2.2)	194 (8.4)	
Missing	3960 (36.4)	3863 (45.1)	97 (4.2)	

Column percentages provided in brackets unless otherwise specified.

* p-P value obtained from chi-squareχ2 test for categorical variables.

BMIbody mass indexHDLhigh density lipoprotein

The average age of sleeplessness-exposed individuals is slightly higher compared with those reporting infrequent or no sleeplessness (unexposed). A greater proportion of exposed individuals were female, smoked daily and had hypertension and dyslipidaemia as compared with those not exposed. Exposed individuals reported lower levels of hard physical activity and educational attainment than unexposed individuals. The overall prevalence of binge drinking did not vary by sleeplessness exposure.

### Description of outcome

The overall incidence risk of diabetes over the study period (1994–2016) was 7.2% (n=784). The incidence risk over the follow-up period among participants who reported experiencing sleeplessness at least once per month at baseline was 8.8%, compared with 6.8% among those who reported infrequent or no sleeplessness. Without adjustment for potential confounders, sleeplessness-exposed participants had 33% greater odds of developing diabetes (OR_unadj_ 1.33, 95% CI 1.13 to 1.57, p<0.001) compared with unexposed participants. The unadjusted association between other covariates and incident diabetes is summarised in online supplemental table 2.

### Multivariable analyses: primary sleeplessness exposure

After adjusting for all confounders, participants who reported sleeplessness at least once a month were found to have 23% greater odds (OR_fully-adj_ 1.23, 95% CI 1.03 to 1.47, p=0.022) of developing diabetes compared with those reporting infrequent or no sleeplessness (table 2). There was no indication of collinearity issues in the final model; large fluctuations in the SE value were not detected during model construction.

**Table 2:** Summary of ORs obtained from unadjusted and adjusted multivariable logistic regression analyses for incident diabetes mellitus by dichotomised sleeplessness exposure and by season-specific sleeplessness exposure

Sleeplessness	Unadjusted model (n=10 875)		Age-, sex-adjusted model (n=10 875)		Fully adjusted model* (n=10 875)	
	OR for DM(95% CI)	P value†	OR for DM(95% CI)	P value†	OR for DM(95% CI)	P value†
Less than once per month	ref		ref		ref	
At least once per month	1.33 (1.13 to 1.57)	<0.001	1.28 (1.08 to 1.52)	0.004	1.23 (1.03 to 1.47)	0.022
	**Unadjusted model (n=10 778)**		**Age-djusted, sex-adjusted model (n=10 778)**		**Fully adjusted model (n=10 778)**	
**Sleeplessness by season**	**OR (95% CI)**	**P value** **†**	**OR(95% CI)**	**P value** **†**	**OR(95% CI)**	**P value** **†**
No sleeplessness	ref		ref		ref	
Sleepless, no particular time	1.45 (1.17 to 1.79)	<0.001	1.38 (1.12 to 1.71)	0.003	1.34 (1.07 to 1.68)	0.010
Sleepless in polar night	1.07 (0.79 to 1.44)	0.660	1.04 (0.77 to 1.41)	0.794	1.03 (0.75 to 1.41)	0.854
Sleepless in midnight sun	1.38 (0.71 to 2.65)	0.340	1.40 (0.72 to 2.71)	0.323	1.59 (0.79 to 3.17)	0.192
Sleepless in Spring/Autumn	1.32 (0.80 to 2.19)	0.279	1.27 (0.76 to 2.12)	0.352	1.06 (0.62 to 1.80)	0.841

* Data in the fully adjusted model waswere adjusted for age, sex, hypertension, dyslipidemiadyslipidaemia, body mass index (), daily smoking, time performing hard physical activity, highest level of education attained, and family history of diabetes. Refer to Supplemental Table S3online supplemental table 3 for full model output for all included variables.

† p-P value obtained from Wald test, H_0_=coefficient=0 (OR=1).

DMdiabetes mellitus

### Multivariable analyses: secondary season-specific sleeplessness exposure

Based on the magnitude of OR point estimates from analyses of season-specific sleeplessness exposure, the association between sleeplessness and incident diabetes is strongest when sleeplessness occurs at no particular time of year (non-seasonal) or during midnight sun (summer), with minimal effect on diabetes incidence due to sleeplessness during polar night (winter) or spring/autumn (table 2); however, the three season-specific findings may be due to chance as the wide CI around the estimates include the null. The OR point estimate for diabetes by specific exposure to non-seasonal sleeplessness is approximately 10% higher than the value for general exposure to sleeplessness from the primary analyses, though the CIs overlap. This relative difference remains even when comparing to a version of the primary model that is restricted to only participants included in the secondary exposure model (n=10 778).

### Stratified and sensitivity analyses

A fully adjusted model with the sex interaction term specified found no difference in the association between sleeplessness and diabetes by sex within the study cohort. Both women and men who reported sleeplessness experienced, on average, 24% (OR 1.24, 95% CI 0.97 to 1.59, p=0.088) and 22% (OR 1.22, 95% CI 0.94 to 1.58, p=0.127) higher odds of diabetes, respectively, compared with those of the same sex who reported infrequent or no sleeplessness. These values are comparable to the unstratified effect measure from the fully adjusted model without sex interaction and there was no evidence of effect modification by sex (p_LRT_=0.944). The OR point estimate for the association between sleeplessness and incident diabetes differed by age group, with the largest effect estimate observed in participants aged 30–39 (OR 1.99, 95% CI 1.40 to 2.83, p<0.001), followed by those under 30 years of age (OR 1.60, 95% CI 0.80 to 3.18, p=0.184). The data yielded no evidence of the association between sleeplessness and diabetes outcome among participants aged 40 or older at baseline (see table 3). Overall, there was some evidence that age modifies the association between sleeplessness and incident diabetes (p_LRT_=0.039) though age group-specific effect estimates are subject to a large degree of uncertainty, as reflected in wide CIs which precludes precise assessment of effect differences between specific strata.

**Table 3:** Summary of ORs for incident diabetes in individuals who experience sleeplessness at least once a month compared with unexposed individuals

	OR for DM (95% CI) among sleepless individuals	Wald p value*	LRT p value†
Unstratified	1.23 (1.03 to 1.47)	0.022	
Sex stratified			0.944
Female	1.24 (0.97 to 1.59)	0.088	
Male	1.22 (0.94 to 1.58)	0.127	
Age stratified			0.039
<30 years	1.60 (0.80 to 3.18)	0.184	
30–39 years	1.99 (1.40 to 2.83)	<0.001	
40–49 years	1.08 (0.80 to 1.45)	0.636	
50–59 years	1.04 (0.74 to 1.48)	0.804	
>60 years	0.88 (0.46 to 1.70)	0.701	

Estimates obtained from fully adjusted age-stratified and sex-stratified multivariable logistic regression analyses.

* p-P value obtained from Wald test, H_0_=coefficient=0 (OR=1).

† p-P value obtained from LRT for interaction by stratifying factor, comparing models with interaction terms specified to those without.

DMdiabetes mellitusLRTlikelihood ratio test

Sensitivity analyses modelling the association between incident diabetes and sleeplessness exposure classified per the original questionnaire response scale yielded OR point estimates consistent with values obtained from the primary analyses modelling sleeplessness as a binary exposure but with a greater degree of uncertainty (table 4). The OR point estimate from the unadjusted model suggests increased strength of association with greater sleeplessness frequency (OR_linear effect_ 1.18, 95% CI 1.09 to 1.28, p<0.001), though the CIs are large and overlap between exposure categories and with the null when exposure was modelled categorically. The evidence for an increasing trend in point estimate was weaker after the association is fully adjusted for other diabetes risk factors (OR_linear effect_ 1.10, 95% CI 1.01 to 1.19, p=0.034). There was no evidence of departure from linearity when modelling sleeplessness effect as a linear trend.

**Table 4:** Summary of ORs obtained from unadjusted, minimally and fully adjusted logistic regression models for the association between sleeplessness categorised per original questionnaire response values and incident diabetes mellitus

Sleeplessness	Unadjusted model (n=10 875)			Age-adjusted, sex-adjusted model (n=10 875)			Fully adjusted model* (n=10 875)		
	OR(95% CI)	P value†		OR(95% CI)	P value†		OR(95% CI)	P value†	
Per original response options (categorical effect model)									
Never or just a few times a year	ref			ref			ref		
1–2 times a month	1.19(0.95 to 1.48)	0.132		1.20(0.96 to 1.51)	0.109		1.22(0.97 to 1.55)	0.094	
Once a week	1.26(0.90 to 1.76)	0.173		1.23(0.87 to 1.72)	0.240		1.18(0.83 to 1.68)	0.356	
>once a week	1.70(1.29 to 2.22)	<0.001		1.47(1.11 to 1.94)	0.006		1.28(0.96 to 1.72)	0.096	
	**OR(95% CI)**	**P value** **†**	**LRT p value** **‡**	**OR(95% CI)**	**P value** **†**	**LRT p value** **‡**	**OR(95% CI)**	**P value** **†**	**LRT p value** **‡**
Per category increment(linear effect model)	1.18(1.09 to 1.28)	<0.001	0.810	1.14(1.05 to 1.23)	0.002	0.832	1.10(1.01 to 1.19)	0.034	0.628

* Data in the fully adjusted model was were adjusted for age, sex, hypertension, dyslipidemiadyslipidaemia, body mass index (), daily smoking, time performing hard physical activity, highest level of education attained, and family history of diabetes. Refer to Supplemental Table S2online supplemental table 2 for full model output for all included variables.

† p-P value obtained from Wald test for categorical effect model estimates, H_0_=coefficient=0 (OR=1).

‡ p-P value obtained from LRT comparing linear effect model to categorical effect model, H_0_=no difference in goodness-of-fit.

LRTlikelihood ratio test

## Discussion

Among Norwegian adults taking part in the Tromsø Study, those who reported experiencing sleeplessness at least once a month had 23% greater odds of developing diabetes compared with those reporting infrequent or no sleeplessness after adjusting for confounders. This finding is supported by sensitivity analyses yielding similar effect point estimates when sleeplessness exposure was classified per the original questionnaire response scale.

The findings of the primary analysis are consistent in direction and magnitude with existing literature^4^,^624 25^ on the association between sleep quality and diabetes among lower latitude (non-Arctic) populations. Secondary analysis of season-specific sleeplessness yielded no evidence that the association of interest was modified by differential exposure to environmental daylight as all estimates had wide CIs overlapping each other and the null. However, a marked difference in the point estimates of association between seasonal categories was observed: a large effect estimate (OR 1.59, 95% CI 0.79 to 3.17, p=0.192) was noted for individuals who were most affected by sleeplessness during summer (midnight sun) with minimal association in the other two season-specific categories. This analysis was hindered by the small number of cases available for each season-specific sleeplessness category and, as such, the current finding may be due to insufficient statistical power rather than a true lack of effect modification. Exposure to artificial light during night-time, as a result of shift work or electronic device usage, has been extensively studied and found to be associated with circadian misalignment, sleep disturbance and, for shift work, increased diabetes risk.^4 26 27^ There is also growing epidemiological evidence linking outdoor light at night to diabetes in the recent literature.^28 29^ Therefore, it may be theorised that circadian misalignment due to natural daylight exposure during the night phase of a circadian rhythm, as occurs during midnight sun, also strengthens the association between the resultant sleeplessness and incident diabetes. Such an interaction aligns with existing theories linking sleep quality to incident diabetes through chronic stress and HPA axis hyperactivation, which are also circadian-dependent physiological processes. Further research focused on season-specific sleeplessness exposure in high-latitude populations would yield valuable information for triangulating the causal relationships between diabetes, sleep quality and light exposure-related circadian misalignment.

Previous studies of sleep quality and incident diabetes in Scandinavian populations,^7^,^9^ including a large-scale, population-based cohort study of Norwegian adults living below the Arctic circle in Nord-Trøndelag county,^9^ failed to find an association among women. The sex-specific estimates of association obtained in this study were surrounded by large CIs that overlap with the null and also cannot be independently interpreted as evidence of an association in either sex. However, statistical analyses (LRT) of our data yielded no evidence that the association differs by sex and it is interesting to note that the consistency between unstratified and sex-stratified point estimates obtained in this study, in addition to the consistency with values from studies in other western European countries^10 11^ and Japan^12^ that report similar or greater strength of association between sleep quality and incident diabetes among women, compared with men. In light of these observations, we suggest that further research is indicated to clarify the role of sex on this association within Scandinavian populations.

### Strengths and limitations

The key strengths of this study include the prospective nature of data collection, long follow-up period, high participation rate and novelty as the first epidemiological investigation of sleep quality and diabetes in an Arctic population. Limitation related to selection and information bias must be noted. The major potential source of selection bias is loss to follow-up that is differential with respect to both sleeplessness and incident diabetes. The data are particularly sensitive to this source of error as over half of Tromsø4 participants were lost to follow-up and did not participate in Tromsø7. Sleeplessness exposure collected at baseline (Tromsø4) reveals that an association exists between sleeplessness and follow-up status—a greater proportion of lost individuals reported experiencing sleeplessness than individuals with complete follow-up. However, no alternative sources of incident diabetes data were available to this study to determine the outcome status of individuals lost to follow-up; as such, it is not possible to accurately specify the association between diabetes outcome and follow-up status. However, it is reasonable to assume that diabetes incidence among those lost to follow-up is likely to be equal to or greater than the rate in study participants as a comparison of baseline data shows that a higher proportion of individuals lost to follow-up had poor health indicators (were daily smokers, had high BMI, hypertension and dyslipidaemia). In this scenario, the error introduced would bias the observed association towards the null, resulting in an underestimate of the true strength of association.

Regarding potential information bias, several aspects of the close-ended question and response set used to elicit self-reported sleeplessness data may have impacted the accuracy and precision of the collected exposure data. While the provided response categories are broadly aligned with the categories used on the Pittsburgh Sleep Quality Index,^30^ the question does not define sleeplessness nor does it differentiate between aspects of poor sleep quality, or between sleep quality and other types of sleep disturbances. The latter is an important consideration for interpreting study findings individually and in comparison to previous studies, as the causal mechanisms leading to diabetes may differ by sleep disturbance type. Additionally, the time frame of reference is not explicitly specified, although one of the responses requires participants to consider frequency up to 1 year in the past; this contrasts with most other methods that reference the past 1–3 months.^5^,^79 31^ These limitations are likely to lead to misclassification of sleeplessness exposure that is non-differential with respect to diabetes outcome as data collection occurs prior to diabetes onset, resulting in some attenuation of the observed effect.

In addition, diabetes detection among participants may have been affected by incomplete HbA1c measurement during Tromsø4 and by the use of self-reported data for incident cases in both surveys. Low HbA1c measurement coverage at baseline may have caused underdetection of diabetes leading to the inclusion of extant diabetes cases that should otherwise be excluded from the study cohort. Additionally, self-reported diabetes and antidiabetic drug use may be less accurate than objective sources such as medical and pharmacy records; though this may not be a significant source of error as self-reported data on diabetes status collected using similar questions has been found to possess high positive and negative predictive value (96.4 and 99.7%, respectively) for identifying diabetes within a Norwegian population.^32^ As neither of the above-listed sources of error are likely to be associated with sleeplessness, the resulting outcome misclassification would be non-differential which would be expected to reduce study efficiency and cause underestimation of the true association.

Important confounders such as presence of sleep-affective comorbidities (eg, depression, stress and anxiety) and ethnicity data were not available; the effect of the latter is partially mitigated by the high degree of ethnic homogeneity in Tromsø^33^ though the potential for information bias from residual confounding by other unmeasured variables remains. In addition, exposure covariates, including sleeplessness, were not tracked for all participants during follow-up as only a subset of Tromsø4 participants were invited to partake in the intervening surveys (Tromsø5/6). Consequently, we were unable to estimate the persistence of sleeplessness among exposed participants nor account for time-varying confounders.

Sleeplessness exposure persistence, or chronicity, is an important consideration when interpreting this study’s findings given the long follow-up period. A high level of persistent sleeplessness among exposed individuals would increase the biological plausibility of an observed association between incident diabetes and sleeplessness measured 20 years earlier because, in such a scenario, exposure measured at a single time point (baseline) is also correlated to cumulative exposure. Longitudinal studies across different geographical populations and age groups have consistently found that insomnia^34 35^ and sleep issues^36^ are persistent conditions. A study tracking Norwegian adolescents observed a chronicity rate of 50.4% for self-reported insomnia symptoms over 6 years of follow-up,^37^ while group-based trajectory modelling of sleep difficulties in working-aged Finnish adults found that steady or worsening trajectories accounted for 52% of all respondents in subgroups with ≥2 nights of sleep difficulties per week at baseline.^38^ It is reasonable to assume that Tromsø residents may experience a similar sleeplessness chronicity rate and consider the biological plausibility of the observed association on this basis.

Green *et al*^39^ observed that the association between cumulative insomnia and incident type 2 diabetes is considerably confounded by other time-varying diabetes risk factors. The authors also demonstrated that traditional logistical regression may fail to adequately account for such confounding bias compared with marginal structural models. Future observational studies should ensure repeated exposure data collection and application of statistical methods suitable for such data to overcome the above-noted limitations. Mendelian randomisation procedures can also be used to address the limitations of traditional observational study designs. This method has already been employed in previous studies among non-Arctic European populations, which found evidence for a causal relationship between insomnia and type 2 diabetes.^40 41^

## Conclusion

Overall, the present findings provide evidence of an association between sleeplessness and incident diabetes among an Arctic population when considered within the context of existing literature and study strengths and limitations. The consistency of our primary analysis results with previous literature adds to the growing body of epidemiological evidence supporting a causal association between sleep quality and incident diabetes. However, further research is required to fully assess whether the association is modified by circadian-effective seasonal daylight variation. This is particularly important as the prevalence of diabetes and sleep problems are expected to continue to rise in the future, and there is increasing need for such data to inform evidence-based development of prevention programmes targeting modifiable risk factors.

## supplementary material

10.1136/bmjph-2023-000644 online supplemental file 1

## Data Availability

Data may be obtained from a third party and are not publicly available.
