# Supplementary material for: Sleeplessness and incident diabetes above the Arctic circle: a secondary analysis of cohort data from the Tromsø Study
Source: BMJ Public Health. 2024 Jan 10;2(1):e000644. doi: 10.1136/bmjph-2023-000644 (PMC11812832; doi:10.1136/bmjph-2023-000644)
Supplement: online supplemental file 1 [file bmjph-2-1-s001.docx]

**Online-Only Supplemental Material**

Rationale for including these additional materials is:

**Figure S1 –** This graphical profile of the Tromsø study surveys is included to provide context and further clarity on the relationship between the surveys used for this study and other surveys carried out as part of the larger, on-going Tromsø study.

**Figure S2 -** DAG is included to summarize theoretical basis for confounder selection in final, fully adjusted model.

**Table S1, S2, S3 –** These data are included to provide readers with the complete results of analyses undertaken in this study, as described in the methods section.

**Supplemental Figure S1.** Graphical summary of seven Tromsø study surveys conducted to-date.

**
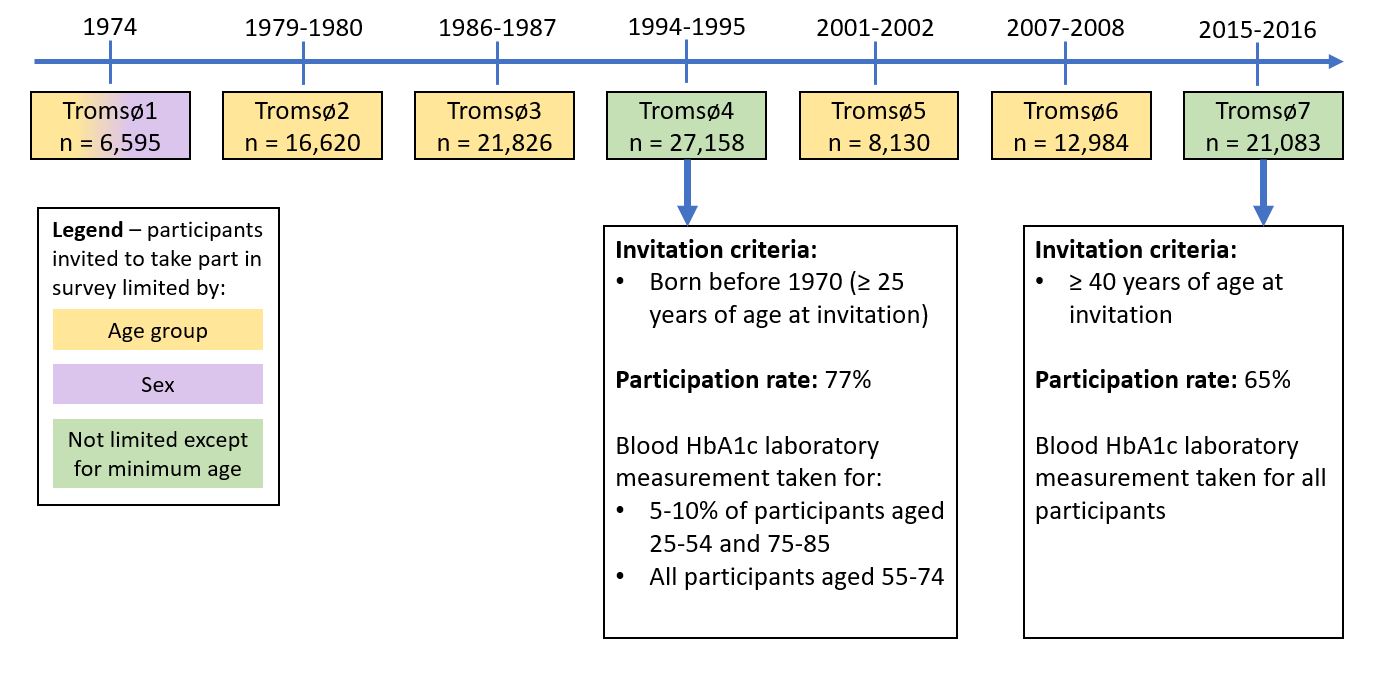
**

**Supplemental Figure S2.** Assumed model of causal relationships between sleeplessness, diabetes mellitus, and other study covariates. Diagram created in Dagitty.


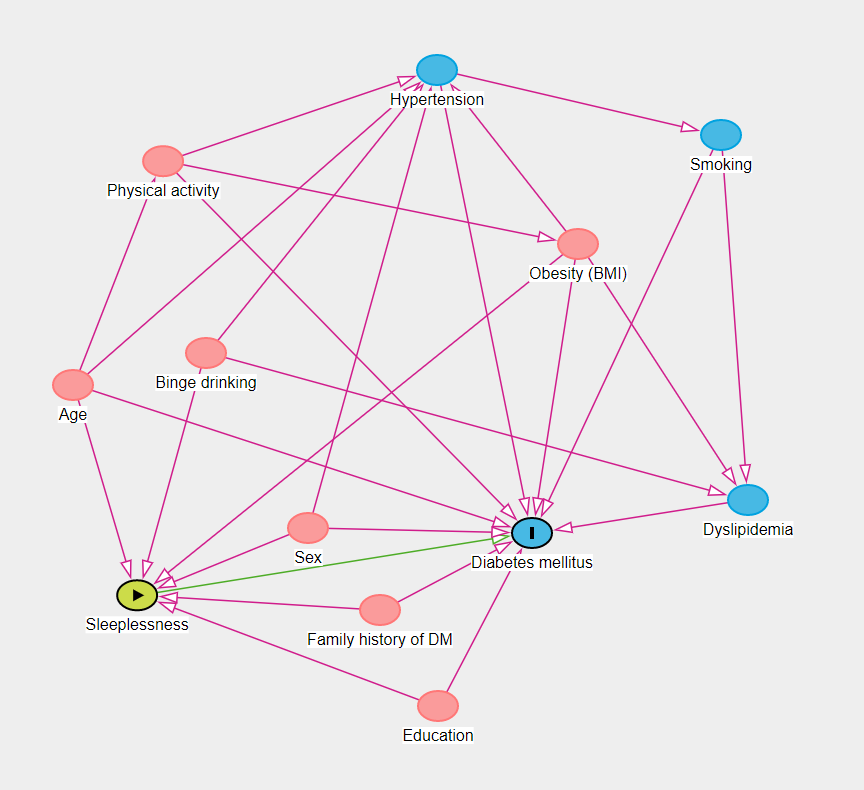


Minimum sufficient adjustment set per above model: Age, Dyslipidemia, Education, Family history of DM, Hypertension, Obesity (BMI), Physical activity, Sex, Smoking

**Supplemental Table S1**. Summary of demographic and behavioural variables of Tromsø4 (T4) participants by follow-up (Tromsø7 (T7) attendance) status.

|  | T4 Total | T4 not followed-up to T7 | T4 followed-up to T7 |
| --- | --- | --- | --- |
| Characteristic at baseline |  |  |  |
|  | n=26,289 | n=13,690 | n=12,599 |
|  |  |  |  |
| Age (years) |  |  |  |
| Mean (SD) | 46.6 (14.9) | 51.2 (17.0) | 41.5 (9.9) |
|  |  |  |  |
| Sex |  |  |  |
| Female | 13,809 (52.5) | 7,073 (51.7) | 6,736 (53.5) |
| Male | 12,480 (47.5) | 6,617 (48.3) | 5,863 (46.5) |
|  |  |  |  |
| BMI |  |  |  |
| < 22 | 5,373 (20.4) | 2,689 (19.6) | 2,684 (21.3) |
| 22 – 23.9 | 5,519 (21.0) | 2,653 (19.4) | 2,866 (22.8) |
| 23.9 – 25.6 | 5,078 (19.3) | 2,534 (18.5) | 2,544 (20.2) |
| 25.6 – 28 | 5,212 (19.8) | 2,754 (20.1) | 2,458 (19.5) |
| > 28 | 5,051 (19.2) | 3,016 (22.0) | 2,035 (16.2) |
| Missing | 56 (0.2) | 44 (0.3) | 12 (0.1) |
|  |  |  |  |
| Blood Pressure |  |  |  |
| Systolic (mmHg), mean (SD) | 134.4 (20.2) | 139.1 (22.6) | 129.2 (15.6) |
| Diastolic (mmHg), mean (SD) | 78.0 (12.4) | 80.0 (13.2) | 75.8 (11.0) |
|  |  |  |  |
| Hypertension  (systolic blood pressure >140 mmHg or diastolic blood pressure > 90 mmHg) |  |  |  |
| No | 17,854 (67.9) | 7,999 (58.4) | 9,855 (78.2) |
| Yes | 8,399 (32.0) | 5,673 (41.4) | 2,726 (21.6) |
| Missing | 36 (0.1) | 18 (0.1) | 18 (0.1) |
|  |  |  |  |
| Blood Lipids |  |  |  |
| Total Cholesterol (mmol/l), mean (SD) (n=11,295) | 6.0 (1.3) | 6.2 (1.4) | 5.8 (1.2) |
| Triglycerides (mmol/l), mean (SD) (n=11,290) | 1.5 (1.0) | 1.6 (1.1) | 1.5 (1.0) |
| HDL (mmol/l), mean (SD) (n=11,282) | 1.5 (0.4) | 1.5 (0.4) | 1.5 (0.4) |
|  |  |  |  |
| Continued… |  |  |  |
|  |  |  |  |
| Table S1 continued | **T4 Total** | **T4 not followed-up to T7** | **T4 followed-up to T7** |
| Characteristic at baseline |  |  |  |
| Dyslipidemia  (non-HDL cholesterol ≥ 4.3 mmol/l) |  |  |  |
| No | 12,208 (46.4) | 5,499 (40.2) | 6,709 (53.3) |
| Yes | 13,982 (53.2) | 8,132 (59.4) | 5,850 (46.4) |
| Missing | 99 (0.4) | 59 (0.4) | 40 (0.3) |
|  |  |  |  |
| Smoke daily |  |  |  |
| No | 16,702 (63.5) | 8,389 (61.3) | 8,313 (66.0) |
| Yes | 9,557 (36.4) | 5,286 (38.6) | 4,271 (33.9) |
| Missing | 30 (0.1) | 15 (0.1) | 15 (0.1) |
|  |  |  |  |
| Hard Physical Activity |  |  |  |
| None | 12,739 (48.5) | 7,747 (56.6) | 4,992 (39.6) |
| < 1 hour/week | 5,384 (20.5) | 2,380 (17.4) | 3,004 (23.8) |
| 1-2 hours/week | 5,256 (20.0) | 2,201 (16.1) | 3,055 (24.3) |
| 3 ≥ hours/week | 2,723 (10.4) | 1,246 (9.1) | 1,477 (11.7) |
| Missing | 187 (0.7) | 116 (0.9) | 71 (0.6) |
|  |  |  |  |
| Binge drinking (past 12 months) |  |  |  |
| Not at all in past year | 7,037 (26.8) | 3,206 (23.4) | 3,831 (30.4) |
| A few times | 8,212 (31.2) | 3,543 (25.9) | 4,669 (37.1) |
| 1-2 times a month | 3,951 (15.0) | 1,773 (13.0) | 2,178 (17.3) |
| 1-2 times a week | 1,116 (4.3) | 551 (4.0) | 565 (4.5) |
| 3 or more times a week | 106 (0.4) | 64 (0.5) | 42 (0.3) |
| Missing | 5,867 (22.3) | 4,553 (33.3) | 1,314 (10.4) |
|  |  |  |  |
| Sleeplessness |  |  |  |
| No | 16,517 (62.8) | 7,616 (55.6) | 8,901 (70.7) |
| Yes | 5,552 (21.1) | 3,144 (23.0) | 2,408 (19.1) |
| Missing | 4,220 (16.1) | 2,930 (21.4) | 1,290 (10.2) |

**Supplemental Table S2**. Summary of odds ratios obtained from univariable logistic regression analyses for incident diabetes mellitus by each study variable for individuals included in primary analysis. DM = diabetes mellitus

|  | N | Odds Ratio for DM (95% CI) | *p*-value* |
| --- | --- | --- | --- |
| Age (per year) | 10,875 | 1.05 (1.04-1.06) | <0.001 |
|  |  |  |  |
| Sex | 10,875 |  |  |
| Female |  | 1.00 (*ref*) |  |
| Male |  | 1.44 (1.25-1.68) | <0.001 |
|  |  |  |  |
| BMI | 10,875 |  |  |
| < 22 |  | 1.00 (*ref*) |  |
| 22 – 23.9 |  | 1.62 (1.14-2.30) | 0.007 |
| 23.9 – 25.6 |  | 2.90 (2.09-4.02) | <0.001 |
| 25.6 – 28 |  | 3.92 (2.85-5.39) | <0.001 |
| > 28 |  | 11.67 (8.63-15.78) | <0.001 |
|  |  |  |  |
| Hypertension  (systolic blood pressure >140 mmHg  or diastolic blood pressure >90 mmHg) | 10,875 |  |  |
| Normal |  | 1.00 (*ref*) |  |
| Hypertension |  | 2.53 (2.17-2.94) | <0.001 |
|  |  |  |  |
| Dyslipidemia (non-HDL cholesterol ≥ 4.3 mmol/l) | 10,875 |  |  |
| No |  | 1.00 (*ref*) |  |
| Yes |  | 1.30 (1.25-1.35) | <0.001 |
|  |  |  |  |
| Smoke daily | 10,875 |  |  |
| No |  | 1.00 (*ref*) |  |
| Yes |  | 1.27 (1.09-1.47) | 0.002 |
|  |  |  |  |
| Hard Physical Activity | 10,875 |  |  |
| None |  | 1.00 (*ref*) |  |
| < 1 hour/week |  | 0.83 (0.69-0.99) | 0.038 |
| 1-2 hours/week |  | 0.59 (0.48-0.72) | <0.001 |
| ≥ 3 hours/week |  | 0.55 (0.42-0.72) | <0.001 |
|  |  |  |  |
| Binge drinking (past 12 months) | 10,591 |  |  |
| Not at all in past year |  | 1.00 (*ref*) |  |
| A few times |  | 0.99 (0.83-1.17) | 0.869 |
| 1-2 times a month |  | 0.96 (0.78-1.18) | 0.691 |
| 1-2 times a week |  | 1.09 (0.77-1.53) | 0.633 |
| 3 or more times a week |  | 2.57 (1.06-6.22) | 0.037 |
| Continued… |  |  |  |
| Table S2 continued |  |  |  |
|  | **N** | **Odds Ratio for DM (95% CI)** | ***p*-value*** |
| Highest Education Level | 10,875 |  |  |
| Primary/partly secondary education |  | 1.00 (*ref*) |  |
| Upper secondary education |  | 0.71 (0.60-0.85) | <0.001 |
| Tertiary education, < 4 years |  | 0.60 (0.48-0.75) | <0.001 |
| Tertiary education, ≥ 4 years |  | 0.45 (0.36-0.56) | <0.001 |
|  |  |  |  |
| Family History of Diabetes | 10,875 |  |  |
| Yes |  | 1.00 (*ref*) |  |
| No |  | 0.40 (0.34-0.46) | <0.001 |
|  |  |  |  |
| Sleeplessness (Primary Exposure) | 10,875 |  |  |
| No |  | 1.00 (*ref*) |  |
| Yes |  | 1.33 (1.13-1.57) | 0.001 |
|  |  |  |  |
| Season-specific Sleeplessness (Secondary) | 10,778 |  |  |
| No sleeplessness |  | 1.00 (*ref*) |  |
| No particular time of the year |  | 1.45 (1.17-1.79) | 0.001 |
| Especially during the polar night |  | 1.07 (0.79-1.44) | 0.660 |
| Especially during the midnight sun season |  | 1.38 (0.71-2.65) | 0.340 |
| Especially in spring and autumn |  | 1.32 (0.80-2.19) | 0.279 |

* p-value obtained from chi-squared test

**Supplemental Table S3**. Summary of ORs obtained from final, fully adjusted multivariable logistic regression primary analysis for incident diabetes mellitus for all included covariates. DM = diabetes mellitus

| n=10,875 | Odds Ratio for DM (CI 95%) | p-value* |
| --- | --- | --- |
|  |  |  |
| Sleeplessness | 1.23 (1.03-1.47) | 0.022 |
|  |  |  |
| Sex |  |  |
| Women | *ref* | - |
| Men | 1.23 (1.04, 1.45) | 0.015 |
|  |  |  |
| BMI |  |  |
| < 22 | *ref* |  |
| 22 – 23.9 | 1.39 (0.97, 1.98) | 0.070 |
| 23.9 – 25.6 | 2.18 (1.55, 3.06) | <0.001 |
| 25.6 – 28 | 2.65 (1.90, 3.70) | <0.001 |
| > 28 | 7.19 (5.22, 9.90) | <0.001 |
|  |  |  |
| Age (per one year increase) | 1.03 (1.02, 1.04) | <0.001 |
|  |  |  |
| Hypertension | 1.37 (1.16, 1.63) | <0.001 |
|  |  |  |
| Dyslipidemia | 1.11 (1.07, 1.16) | <0.001 |
|  |  |  |
| Smoke Daily | 1.50 (1.27, 1.77) | <0.001 |
|  |  |  |
| Education |  |  |
| Primary/partly secondary education | *ref* |  |
| Upper secondary education | 0.95 (0.78, 1.15) | 0.581 |
| Tertiary education, < 4 years | 0.88 (0.69, 1.11) | 0.267 |
| Tertiary education, ≥ 4 years | 0.85 (0.67, 1.08) | 0.192 |
|  |  |  |
| Hard Physical Activity |  |  |
| None | *ref* |  |
| < 1 hour/week | 0.93 (0.77, 1.13) | 0.480 |
| 1-2 hours/week | 0.78 (0.63, 0.96) | 0.020 |
| 3 ≥ hours/week | 0.72 (0.54, 0.96) | 0.027 |
|  |  |  |
| No Family History of Diabetes | 0.43 (0.40, 0.50) | <0.001 |

*p-values obtained from Wald test, H_0_ = coefficient=0 (OR=1)
